# Challenges in the diagnosis and management of immune-mediated necrotising myopathy (IMNM) in a patient on long-term statins

**DOI:** 10.1007/s00296-022-05230-0

**Published:** 2022-10-19

**Authors:** Faris Khan, Stefen Brady, Anoop Kuttikat

**Affiliations:** 1 School of Clinical Medicine, University of Cambridge, Cambridge, CB2 0SP UK; 2 Oxford University Hospitals NHS Foundation Trust, John Radcliffe Hospital, Oxford, OX3 9DU UK; 3 Kettering General Hospital, Kettering, NN16 8UZ UK; 4 Addenbrookes Hospital, Cambridge, CB2 0QQ UK

**Keywords:** Muscle weakness, Creatine kinase, Immunoglobulin intravenous, Methotrexate, Immunosuppressive agents, Prednisolone

## Abstract

Immune-mediated necrotising myopathy (IMNM) is a severe and poorly understood complication of statin use. Prompt management with immunosuppressive treatment is often needed to control the condition, which differs from the management of the more commonly recognised statin-induced myopathy. We present a case report and brief review of the literature regarding the pathogenesis, diagnosis, and management of anti-3-hydroxy-3-methylglutaryl-coenzyme A reductase (HMGCR) positive IMNM (HMGCR IMNM). There are no randomised clinical trials, but several smaller studies and cases suggest a triple therapy of corticosteroids, IVIG, and a corticosteroid-sparing immunosuppressant appears efficacious in patients with IMNM and proximal weakness. The mechanism of statin-induced IMNM is uncertain, and this is further complicated by the reports of HMGCR IMNM in statin-naïve patients, including children. We present a case of biopsy-confirmed HMGCR IMNM in a woman taking daily statins for treatment of hypercholesterolaemia for 4 years. She presented with symptoms consistent with a urinary tract infection (UTI), including muscle weakness. She was treated as an isolated case of UTI. One month later, she presented again with worsening weakness in her shoulders and hips. Creatine kinase was elevated, and MRI showed increased signal with STIR sequences in both thighs. Anti-HMGCR was positive and leg biopsy-confirmed necrotising changes. Stopping her statin prescription and a short course of prednisolone did not improve her muscle weakness. Adding methotrexate resulted in eventual resolution of her symptoms. IMNM should be considered as a differential in any patient taking statins presenting with muscle weakness, and this case suggests that immunosuppressant therapy in addition to cessation of statins is effective at treating IMNM. Clinical trials are needed to further investigate the efficacy of different combinations of immunosuppressants.

## Introduction

Immune-mediated necrotising myopathy (IMNM) is a rare type of autoimmune myopathy characterised by relatively severe proximal weakness and is commonly associated with the development of anti-HMGCR autoantibodies in older adults who are taking statin medication.

The first cases of anti-HMGCR antibody-associated IMNM were identified in 2010 by serum screening for novel immunospecificities in patients who, at the time, were defined as “autoantibody-negative” [1]. HMGCR catalyses the conversion of (3S)-hydroxy-3-methylglutaryl-CoA (HMG-CoA) to mevalonic acid, the rate-limiting step in the synthesis of cholesterol and other isoprenoids, and thus plays a critical role in cellular cholesterol homeostasis. HMGCR is the primary target of statins, the main class of cholesterol-lowering agents. IMNM must be distinguished from other myopathies, including non-statin-related idiopathic inflammatory myopathy (IIM) and late onset muscular dystrophy. Previous classifications relied on muscle biopsy [2]. However, muscle biopsies were not found to be specific or sensitive for the various forms of myositis. IMNM must also be differentiated from other statin-induced myopathies, such as rhabdomyolysis, myopathy, and rhabdomyolysis. In 2017, the European Neuromuscular Centre identified three diagnostic criteria for IMNM; the presence of elevated CK levels, proximal muscle weakness, and anti-HMGCR autoantibodies or anti-SRP autoantibodies. These diagnostic criteria are not well known in primary care and emergency settings, and this can lead to delays in diagnosis of this debilitating condition, as seen in our patient. Increasing awareness of this condition may lead to faster referral of patients presenting with muscle weakness for CK levels and autoantibody screens.

The prevalence of IMNM is not fully known. The incidence of statin-induced myopathies based on RCTs of statin treatment is 1.5–5.0% [3]. Furthermore, the prevalence of autoimmune myopathies, of which IMNM is a subtype, is just 9–14 cases per 100,000 adults, and only about 6% have anti-HMGCR antibodies [4, 5]. Over 90% of cases of HMGCR IMNM appear to be in adults who are taking statin medication [5]. There are currently no RCTs focussing on IMNM, further highlighting the rarity of this condition.

Recently, we encountered a patient on long-term statins with no myositis-related history and unspecific clinical symptoms of muscle weakness, who was initially misdiagnosed and only correctly diagnosed with HMGCR IMNM following worsening of clinical symptoms. Delayed diagnosis can have significant impacts on a patient’s life, including on their mental health. With the ever-increasing use of statins, we expect a significant increase in presentations of even the rarest side-effects, such as IMNM, and thus, improved awareness for the diagnosis and management of this condition is important [6].

## Case report

**Fig. 1:**
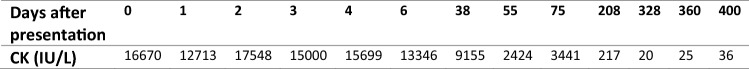
Creatine Kinase (CK) levels at various stages after initial presentation

In June 2020, a 51-year-old female with a 5 year history of hypercholesterolaemia, hypertension, diabetes mellitus, and frequent recurrent urinary tract infections initially presented to her general practitioner (GP) with concerns over increasing difficulty climbing stairs. She had been taking 20 mg Atorvastatin oral once daily (OD) since 2016 as a result of her hypercholesterolaemia. No prior history of similar episodes of muscle weakness was noted by her GP. Around the same time as her onset of weakness, she developed lower abdominal pain and was found on urinalysis to have a urinary tract infection, with symptoms of increased urinary frequency and dysuria), and following a urine culture that confirmed *Escherichia coli* (> 10^5). Microbiology analysis indicated that infection was nitrofurantoin sensitive. With a working diagnosis of a lower urinary-tract infection being the cause of her presentation, GP advised urgent antibiotic treatment and she was commenced on nitrofurantoin (50 mg 4 times a day for 3 days, in accordance with British National Formulary, guidelines for a lower urinary-tract infection). Despite improvement in her urinary symptoms, she continued to get weaker, particularly around her shoulders and legs. She was referred to hospital by her GP in July 2020, and a blood test showed a raised creatinine kinase (CK) of 16,670 iu/L (normal range 22 to 198 iu/L), prompting admission (Fig. 1). Her atorvastatin was stopped at admission.

Examination revealed mild weakness (Muscle Research Council Score [MRC] 4/5) of neck flexion, moderate weakness (MRC 4/5) of shoulder abduction, and severe right (MRC 2/5) and moderate left (MRC 3/5) hip flexion weakness. She walked with Trendelenburg gait, suggesting week hip abductors, and her triceps and ankle jerk reflexes were absent bilaterally. No skin lesions were identified.

**Fig. 2:**
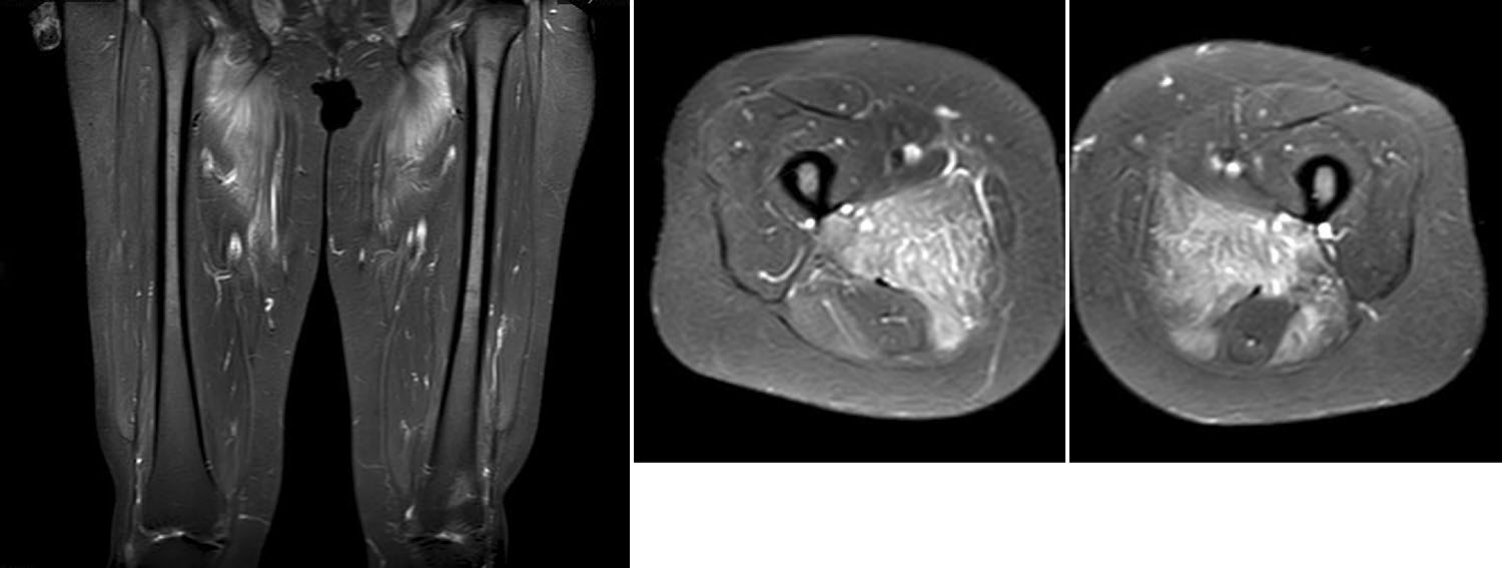
Bilateral T2-weighted MRI of the patient’s thighs showing extensive muscle oedema bilaterally involving posterior thigh muscle groups, with relative sparing of the semitendinosus muscles bilaterally. These MRI results were consistent with bilateral inflammatory changes, akin to those seen in polymyositis. Left: coronal MRI; right: axial MRI

A bilateral leg magnetic resonance imaging (MRI) was performed, and a muscle biopsy was taken as part of an extensive diagnostic workup. MRI of her thighs showed extensive muscle oedema bilaterally involving posterior thigh muscle groups, with relative sparing of the semitendinosus muscles bilaterally (Fig. 2). There was also involvement of the gluteus maximus muscles, adductor muscles, obturator internus muscles, and iliopsoas muscles bilaterally. These MRI results were consistent with bilateral inflammatory changes, akin to those seen in polymyositis. As a result, the patient was started on intravenous (IV) methyl prednisolone 500 mg for 3 days, followed by oral prednisolone 60 mg once daily.

**Fig. 3:**
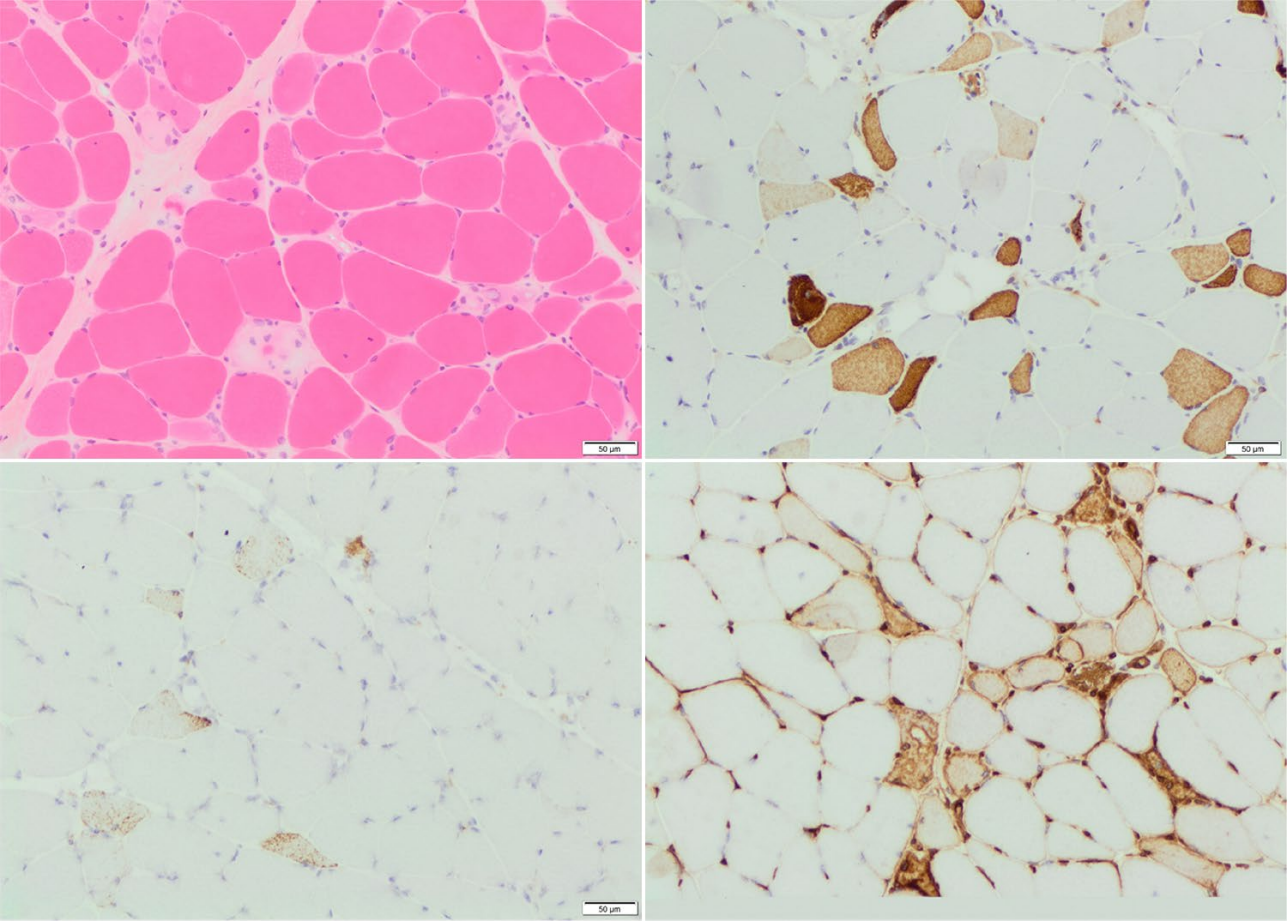
Biopsy images of a patient with anti-HMGCR IMNM. Top Left: photomicrograph of H&E-stained frozen section; scattered fibres observable at various stages of necrosis. Regeneration not so evident in this image but present elsewhere. Relative lack of inflammation. Top right: immunostain for fetal (neonatal) myosin; scattered positive fibres. Fetal myosin no longer present in normal muscle after 1 year of age. Can depict necrotic/regenerating fibres in this context. Bottom left: immunostain for p62; finely granular punctate staining in scattered fibres. Characteristic but not entirely specific for IMNM. Bottom right: immunostain for MHC-1; focal sarcolemmal and sarcoplasmic upregulation. In normal muscle, MHC-1 stains blood vessels/capillaries, as seen here (positive internal control)

Muscle biopsy taken from a region of oedema present in the thigh muscle was consistent with necrotising myopathy (Fig. 3). CT chest, abdomen, and pelvis were performed to rule out paraneoplastic causes, and were unremarkable. Routine autoimmune blood tests were also performed, as well as anti-HMGCR test, since she had been taking atorvastatin (Table 1). The patients’ anti-HMGCR antibodies were raised at 191.90 CU (reference range 0–20 CU). The combination of positive anti-HMGCR and necrotising changes on biopsy led to the diagnosis of immune-mediated necrotising myopathy. She was discharged home on 60 mg of prednisolone.

**Table 1:** Auto-antibody screen results

Antibody	Test result
Anti-nuclear	Negative
Anti-dsDNA	Negative
Extractable nuclear antigens (anti-Ro, -La, -Sm, -RNP, -Scl-70)	Negative
Myositis specific (anti-TIF1-γ and anti-Jo)	Negative

**Table 2:** Summary of previous cases of HMGCR IMNM in patients on long-term statins, including age, sex, duration of statin use prior to presentation, diagnostic methods, and management details

Authors	Year	Age/gender	Statin use (years)	Diagnosis	Management	Recovery (months)	Relapse	Additional
Lim et al. [7]	2020	54/M	1	Symptoms HMGCR + biopsy	MethylPDN, PDN, MTX, IVIG, HCQ	6	No	Presented with DM-like cutaneous features
Piedra et al. [8]	2020	73/M	> 1	Symptoms HMGCR + biopsy	PDN, MethylPDN, MTX, IVIG, AZA	N/A (died in hospital)	N/A	MTX stopped due to elevated LFTs AZA discontinued due to infection
Paul et al. [9]	2021	43/F	5	Symptoms HMGCR + biopsy	PDN, IVIG	5	No	PDN alone for 8 weeks with little response
Stroie et al. [10]	2020	59/M	> 1	Symptoms HMGCR + biopsy	PDN, AZA, IVIG	4	Yes	Initially PDN alone, relapsed on tapering
Joudeh et al. [11]	2022	55/M	2.5	Symptoms HMGCR + EMG biopsy	MethylPDN, PDN, IVIG, RTMB	7	Yes	Recurrent relapses of muscle weakness
Sharma et al. [12]	2019	71/M	> 10	Symptoms HMGCR + EMG biopsy	MethylPDN, PDN, IVIG, AZA	> 1	No	Statin use only stopped after muscle biopsy results
Lahaye et al. [13]	2014	65/M	2	Symptoms HMGCR + biopsy	PDN, MTX, IVIG	12	No	On MTX and PDN for 12 months
Zhang et al. [14]	2019	68/M	3	Symptoms HMGCR + biopsy	PDN, MTX, CLSP, IVIG	7	Yes	Relapsed after 4 years following weaning of MTX and PDN. Subsequent IVIG, MTX and RTMB for 3 years for recovery
		62/M	3	Symptoms HMGCR + biopsy	PDN, MTX, CLSP, IVIG, TPX, RTMB	4	Yes	Frequent relapses for one year until RTMB added, still on RTMB

Methylprednisolone (methylPDN), prednisolone (PDN), methotrexate (MTX), intravenous immunoglobulin (IVIG), hydroxychloroquine (HCQ), dermatomyositis (DM), azathioprine (AZA), electromyography (EMG), rituximab (RTMB), cyclosporine (CLSP), therapeutic plasma exchange (TPX)

The patient remained on 60 mg prednisolone for a total of 3 weeks, followed by 2 weeks of dose tapering, with no significant change in symptoms, and on 6-week follow-up, the patient had no significant change in her symptoms, and continued to struggle climbing stairs. She was started on methotrexate, building up to 15 mg PO once weekly. On follow-up in late-November 2020, 3 months after commencing methotrexate, examination revealed mild improvement, with moderate weakness on neck flexion when supine (MRC 4/5), mild weakness with shoulder abduction (MRC 4/5), and mild weakness of hip flexion (MRC 4/5). It is unlikely these effects were nitrofurantoin related as the treatment duration was very short (3 days), and nitrofurantoin has not been implicated in anti-HMGCR myositis previously. On assessment 7 months later, the patient continued to improve and was able to rise from a chair without using her arms and climb the stairs. 18 months after initial presentation, the patient reported no longer having any symptoms (MRC 5/5 in all muscle groups).

## Search strategy

**Fig. 4:**
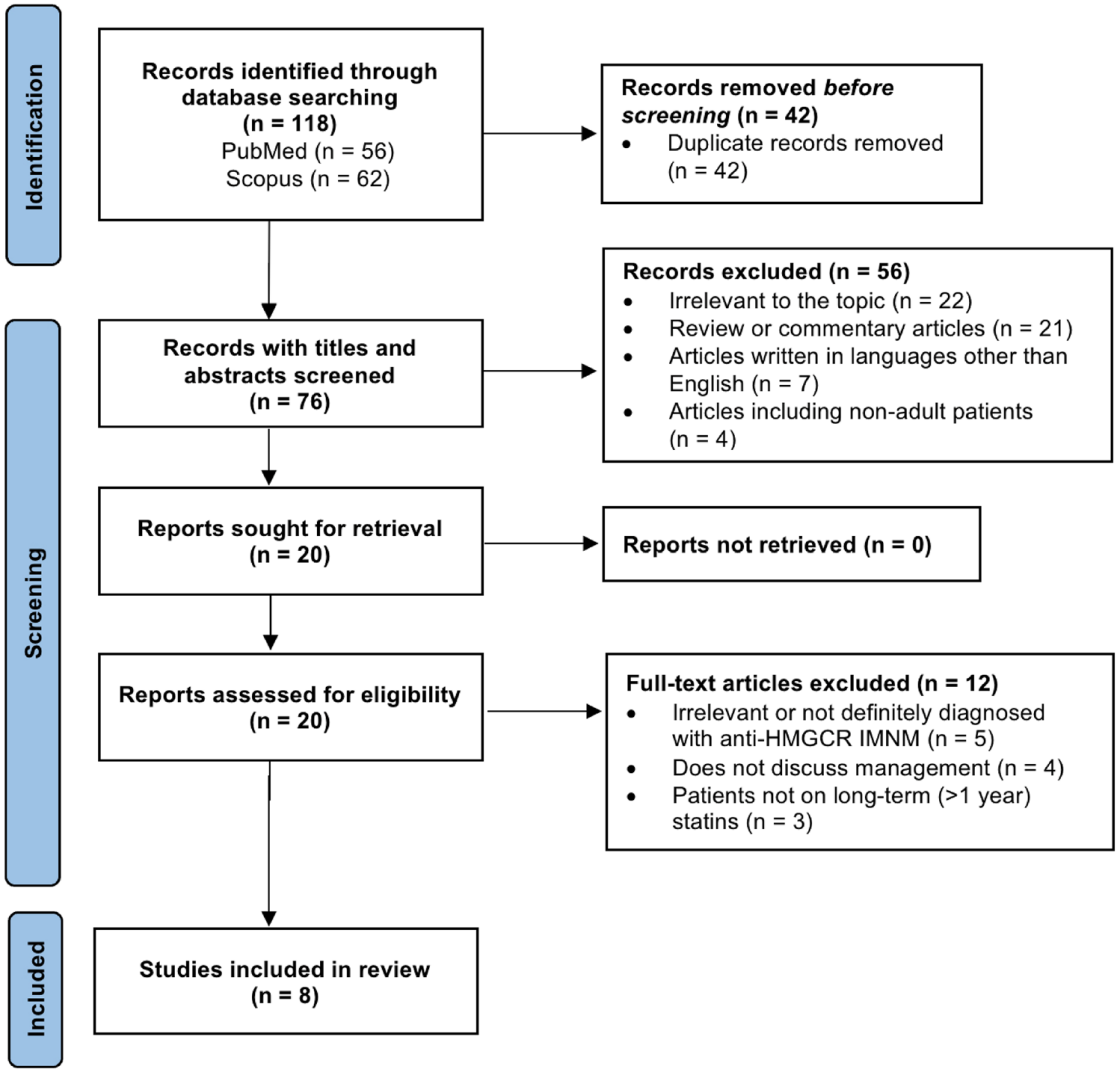
Flow diagram of the literature review

We searched MEDLINE/PubMed and Scopus for articles published by August 2022, with the key words: “HMGCR” AND “immune-mediated necrotizing myopathy” AND “statin”. We identified 118 articles, and relevant references were carefully reviewed by one author (FK) for eligibility (Fig. 4). We excluded articles that were written in languages other than English, irrelevant articles, duplicates, reviews and commentary articles, articles involving non-adult patients, and articles that did not specifically report on the management of HMGCR IMNM in patients on long-term statins (defined as > 1 year of statin use prior to presentation). We identified 8 original articles of HMGCR IMNM associated with long-term statin use (Table 2).

## Discussion

HMGCR IMNM comprises myositis with positive autoantibodies against the enzyme HMGCR. HMGCR is involved in the cholesterol biosynthesis pathway. Data by Christopher-Stine et al. suggest that the development of these enzymes is highly correlated with statin exposure. It is unclear how the presence of anti-HMGCR antibodies can lead to selective, prolonged muscle necrosis in some individuals [1]. One hypothesis is the expression of HMGCR in the sarcolemma of some patients with IMNM, along with expression of complement components. Therefore, the muscle necrosis may be the result of antibody-dependent complement activation [15]. There may be a genetic predisposition in some individuals towards the expression of HMGCR in muscle sarcolemma [16]. Furthermore, some patients with HMGCR IMNM have never received statins; the prevalence of statin naïve INNM appears to vary greatly with region. This often involves younger patients. The prognosis of these patients is worse, with symptoms harder to treat with immunosuppressants. Therefore, it is likely there are other genetic or environmental factors that are associated with the development of IMNM. Genetic links have been identified in the form of some major histocompatibility complexes, for example, HLA-DRB1 which is associated with the development of anti-HMGCR antibodies [17]. In high-risk patients (for example, those with a family history of statin-related myopathies), it may be beneficial to investigate prescribing alternative cholesterol-lowering medications as first line, to avoid the detrimental effects that these myopathies can bring to a patient. Some pharmacological statin-naïve cases of IMNM may also be due to alimentary sources of statins, for example, cases in Asia [4].

HMGCR IMNM differs from the more common statin-induced myopathy, and importantly, from a clinical perspective, IMNM symptoms fail to resolve after discontinuation of the offending drug, consistently highlighted in case reports [4]. Statin-induced myopathy is a well-reported adverse effect of statin use, but increasing awareness of HMGCR IMNM is essential in, for example, primary care to allow for appropriate testing and subsequent management in this challenging patient group. Treatment of anti-HMGCR myopathy appears to be safer and more efficacious if commenced early, perhaps due to lower doses and durations of immunosuppressant required, and more minimal inflammation in the early stages of diseases [18]. Our review of the literature suggests that in general, a triple therapy of corticosteroids, IVIG, and a corticosteroid-sparing immunosuppressant appears efficacious in patients with proximal weakness [7–14]. However, a dual combination of steroids and a steroid-sparing agent may be sufficient, especially important as there is a global shortage of IVIG. Our patient was not started on combination therapy, and was instead trialled on initial IV steroids, then oral corticosteroids for 1 month, both with minimal improvements in strength. Only after adding methotrexate did the patients’ health begin to improve.

There is still great uncertainty regarding the optimum management regime for these patients. With our patient, 4 weeks of steroids may not have been sufficient time for significant improvements, if any, and the addition of methotrexate may have turned the tide and led to symptom improvement, but it may also have just been the continued steroid use. Nonetheless, delaying recovery can have detrimental effect on the patients’ physical and social health. Our patient was exposed to high doses of steroids for a considerable duration. Being on long-term steroids brings its own side-effects and can affect patient comorbidities, including glucose tolerances in diabetic patients [19]. Diabetes is a common comorbidity in this group of patients taking statins [20]. Arguments can be made in support of the judicious use of high-dose steroid as part of an effective (and cheap) treatment strategy that ultimately improves clinical outcomes by helping the patient into remission as quickly as possible. Nonetheless, tapering down the dose of steroid as quickly as possible can help to reduce side-effects, but may lead to relapse [10, 14]. Awareness is one aspect that can lead to improved patient outcomes, and further controlled studies are required to identify the optimal choice and timing of treatment agents.

Relapse is common in this group of patients with tapering of medication [10, 11, 14]. In her most recent follow-up, our patient was yet to experience any symptoms associated with relapse. In our patient, and the 8 articles we reviewed, improvements in symptoms (and therefore, disease recovery) correlated with a drop in CK levels. Therefore, CK levels are a good indicator of disease level and CK blood tests are readily available in primary care, and levels may be a useful marker to utilise in future consultations if there are concerns of relapse. Further studies are essential to determine the optimum method of medication withdrawal to minimise the risk of relapse. It is also important for clinicians to realise that the presence of IMNM autoantibodies alone is not specific for myositis and, based off current research, there would be little benefit to anti-HMGCR antibody screening in patients as a way of identifying high-risk individuals [21].

Understanding statin-related side-effects is important as muscle pain increases the likelihood of statin discontinuation and non-adherence, ultimately increasing the risk of major cardiovascular events in this group of patients [22]. IMNM is the most severe and rarest documented form of statin-related myotoxicity (SRM). Some factors that increase statin exposure in vivo include gender and age, and many other non-modifiable and modifiable risk factors that affect the absorption, distribution, metabolism, and elimination of statins. The PRIMO study provided important information regarding SRMs. Interestingly, 85% of muscle symptoms begun within 1 month of statin initiation, or titration to a higher dose [3]. It is therefore relatively rare to only develop muscle weakness symptoms over 4 years after starting statins, as was the case and those analysed in the review [7–14]. As such, on top of risk factors that influence the development of SRMs such as IMNM, other genetic factors may be at play.

This patient was suffering with an *E. coli* UTI at the same time as the onset of her muscle weakness. It has been reported that myositis can by triggered by various infections, such as UTIs [23]. There is one reported case of anti-HMGCR myositis following acute Epstein–Barr virus infection [24]. There is currently no research on any interaction of bacterial infections with SRMs or IMNM. Recent research has found that statins can interact with the immune system in many ways and one possible mechanism of interaction with infection may be through isoprenoid depletion which may cause a loss of immunological tolerance and cross-reaction between bacterial and human isoprenoid pathways [25–27]. Further studies looking at the specific risk factors or environmental factors involved in the development of HMGCR autoantibodies are needed to fully inform patients on the risks associated with statin us, including the development of IMNM.

It may be worthwhile making CK tests more frequent in primary care in patients taking statins, as CK is generally a good indicator of disease prognosis and is more readily available than anti-HMGCR levels [28, 29]. Currently, NICE only recommends CK testing before starting statin treatment (National Institute for Health and Care Excellence [NICE], 2016) [30]. In our patient, there may have been a delay in diagnosis due to a concomitant UTI. In patients reporting weakness on a statin, it is important to consider the possibility of HMGCR IMNM even if more common presentations like a UTI are thought more likely. CK levels could have been checked alongside routine UTI screening to reliably rule out IMNM.

## Conclusion

HMGCR IMNM is unique amongst SRMs in that it does not improve with statin discontinuation alone, and usually requires a combination of steroid and steroid-sparing immunosuppressant. Early diagnosis is crucial as commencing treatment early leads to better outcomes in patients. Anti-HMGCR antibodies are highly specific for this condition and not associated per se with hyperlipidaemia, self-limiting statin side-effects, or genetic muscle disease. In patients presenting with muscle weakness who are taking statins, it is beneficial to perform anti-HMGCR antibody test (especially if persistent symptoms despite statin cessation) to promptly differentiate this rare condition from more common but less severe statin-related myotoxicities. Randomised clinical trials are required to guide therapeutic decisions in IMNM.

## Data Availability

Data sharing is not applicable to this article as no datasets were generated or analysed during the current paper. The dataset supporting the conclusions of this paper is shown within the article and no additional data could be shared publicly due to patients’ privacy.
